# Decreasing methicillin-resistant *Staphylococcus aureus* (MRSA) infections is attributable to the disappearance of predominant MRSA ST239 clones, Shanghai, 2008–2017

**DOI:** 10.1080/22221751.2019.1595161

**Published:** 2019-03-29

**Authors:** Yingxin Dai, Junlan Liu, Wei Guo, Hongwei Meng, Qian Huang, Lei He, Qianqian Gao, Huiying Lv, Yao Liu, Yanan Wang, Hua Wang, Qian Liu, Min Li

**Affiliations:** Department of Laboratory Medicine, Renji Hospital, School of Medicine, Shanghai Jiaotong University, Shanghai, People’s Republic of China

**Keywords:** *Staphylococcus aureus*, MRSA, antibiotics resistance, clonal shift, sequence types, hospital-acquired infections

## Abstract

A consistently decreasing prevalence of MRSA infections in China has been reported, however, the underlying mechanism of molecular processes responsible for this decline in MRSA infections has been poorly understood. We conducted an epidemiologic investigation to determine the dynamic changes of *Staphylococcus aureus* infections. A total of 3695 *S. aureus* isolates was recovered from 2008 to 2017, and subsequently characterized by infection types, resistance profile, and clone types. The frequency of respiratory infection decreased over the study period from 76% to 52%. The proportion of MRSA remarkably decreased (from 83.5% to 54.2%, 2008-2017) (*p* < .0001). The prevalence of predominant healthcare-associated MRSA (HA-MRSA) clones, ST239-t030 and ST239-t037, significantly decreased (from 20.3% to 1% and 18.4% to 0.5%, 2008-2017, respectively); both of them were replaced by the continually growing ST5-t2460 clone (from 0% to 17.3%, 2008-2017). Epidemic community-acquired MRSA (CA-MRSA) ST59 and ST398 clones also increased (from 1.0% to 5.8% and 1.8% to 10.5%, 2008-2017, respectively). These results demonstrated a significant decrease in the previously dominant HA-MRSA ST239 clones, leading to a marked decrease in the prevalence of MRSA over the past decade, and shed new light on the complex competition of *S. aureus* clones predominating within the health-care environment.

## Introduction

1. *Staphylococcus aureus*, especially MRSA, has been considered as a crucial pathogen posing a serious threat to public health for the past decades [1]. The mean prevalence of MRSA across China was over 50% in 2005, whereas it was much higher in Shanghai [2]. As the primary cause of lower respiratory tract infections and surgical site infections [3, 4], the infections caused by *S. aureus* are refractory due to its evolved resistance to antimicrobial drugs [5] *S. aureus* is also infamous for its multiple virulence factors contributing to various diseases [6]. However, virulence genes differ in existence and expression among different genotypes, that is, *S. aureus* of specific molecular type will be closely related to specific infection types [7].
2. *S. aureus* has been undergoing rapid evolution by horizontal gene transfer [8], thus diverse molecular techniques have been widely applied to monitor the evolutionary process of the emergence and geographical spread of pandemic clones [9]. It is vital to investigate the changing epidemiology of *S. aureus* infection over a long period for the purpose of guiding new control initiatives, providing keen insights into its evolutionary process, and containing its spread. While numerous studies have reported epidemiological changes in HA-MRSA [10], few studies have focused on the changing epidemiology of both MRSA and methicillin-sensitive *S. aureus* (MSSA) isolates over a long period of time in China, despite the fact that MSSA isolates provide a pool of organisms for the emergence of new MRSA clones, and are of essence for controlling the potential emergence of new epidemic MRSA clones [11]. Hence, the present study retrospectively characterized *S. aureus* isolates involved in hospital-onset infection over the past decade in a tertiary care hospital, one of the largest comprehensive hospitals in Shanghai, and aimed at investigating the changes in the molecular characteristics of both MRSA and MSSA, as well as the pattern of infection type and antibiotic resistance profile.

## Materials and methods

*Bacterial isolates*. A total of 3695 consecutive and non-repetitive *S. aureus* isolates was collected from a comprehensive teaching hospital in Shanghai, China between 2008 and 2017. All of the inpatients in this study acquired *S. aureus* infection after hospital admission. In addition to routine microbiology/biochemical methods (e.g. Gram staining and catalase and coagulase activity on rabbit plasma), MALDI-TOF/MS was used to further confirm identities of the *S. aureus* isolates. All isolates were stored at −80°C for later use.

*Antimicrobial susceptibility testing*. The antimicrobial susceptibility of all isolates in this study was evaluated using the Biomerieux Vitek 2 system following manufacturer’s instructions. Results were interpreted in accordance with Clinical and Laboratory Standards Institute (CLSI) guidelines. The antimicrobial agents tested included cefoxitin, penicillin, gentamicin, cefazolin, erythromycin, fosfomycin, rifampin, trimethoprim/sulfamethoxazole, levofloxacin, linezolid, and vancomycin. *S. aureus* ATCC29213 was used as a quality control.

*Molecular typing methods*. Chromosomal DNA was extracted following culture on blood agar plates by a standard phenol–chloroform extraction procedure and used as a template for PCR reaction. Multi-locus sequence typing (MLST) was carried out according to the method as previously described [12]. The following housekeeping genes were detected: carbamate kinase (*arcC*), shikimate dehydrogenase (*aroE*), glycerol kinase (*glp*), guanylate kinase (*gmk*), phosphate acetyltransferase (*pta*), triosephosphate isomerase (*tpi*), and acetyl coenzyme A acetyltransferase (*yqiL*). The sequences of the PCR products were compared with the existing sequences available on the MLST website (http://www.pubmlst.net) for *S. aureus*, and the alleles of the seven genes define the *S. aureus* lineage, resulting in an allelic profile designated sequence type (ST). *spa* typing was carried out by amplification and sequencing of polymorphic X region of the protein A gene (*spa*), then *spa* types were assigned by using the *spa* database website (https://www.spaserver.ridom.de).

*Statistical analysis*. All statistical tests were performed with the GRAPHPAD software system. Percentage values or frequencies were analysed pairwise by two tailed chi-square test or Fisher’s Exact Test. Linear trends were tested using linear regression or the chi-square test for trend, and a *p*-value <.05 was considered statistically significant.

## Results

### Demographic characteristics of hospital-onset *S. aureus* infections, 2008–2017

A total of 3695 consecutive and non-repetitive *S. aureus* isolates was collected between 2008 and 2017 from inpatients, most of whom were aged over 20 years old (Table 1). When stratified by age, a small but statistically significant increase was observed in the proportion of the group aged ≥65 years (from 47% in 2008 to 56% in 2017; *p* < .0001); in contrast, the proportion of inpatients ranging from 21 to 64 years old was reduced significantly from 50% (2008) to 38% (2017) (*p* = .0036) (Table 1). Moreover, the vast majority of *S. aureus* isolates were recovered from male rather than female (Table 1). This significant differential in gender distribution indicated that males were still more susceptible to *S. aureus* than female, even though the proportion of male inpatients was greatly decreased in 2017 compared to 2008 (73 vs. 66%; *p *<* *.01).

**Table 1.** Dynamic changes in specimen sources and basic demographics of inpatients with infection caused by *S. aureus* from 2008 to 2017.

Year	2008	2010	2011	2012	2015	2016	2017
No. of total	770	495	608	462	513	426	421
Gender							
Male/female	73%/27%	70%/30%	69%/31%	65%/35%	73%/36%	70%/30%	66%/34%
Age group							
≤20	4%	5%	2%	5%	3%	4%	7%
21–64	50%	45%	44%	35%	43%	38%	38%
≥65	47%	50%	54%	60%	54%	58%	56%
Specimen sources							
Respiratory system	588(76%)	311(63%)	420(69%)	261(56%)	317(62%)	238(56%)	218(52%)
Skin/soft tissue	93(12%)	107(22%)	89(15%)	108(23%)	97(19%)	107(25%)	112(27%)
Blood	21(3%)	22(4%)	29(5%)	22(5%)	29(6%)	25(6%)	24(6%)
Other sterile body fluids	43(6%)	41(8%)	70(12%)	41(9%)	62(12%)	47(11%)	45(11%)
Others	25(3%)	14(3%)	0(0%)	30(6%)	7(1%)	9(2%)	22(5%)

**Figure 1.**
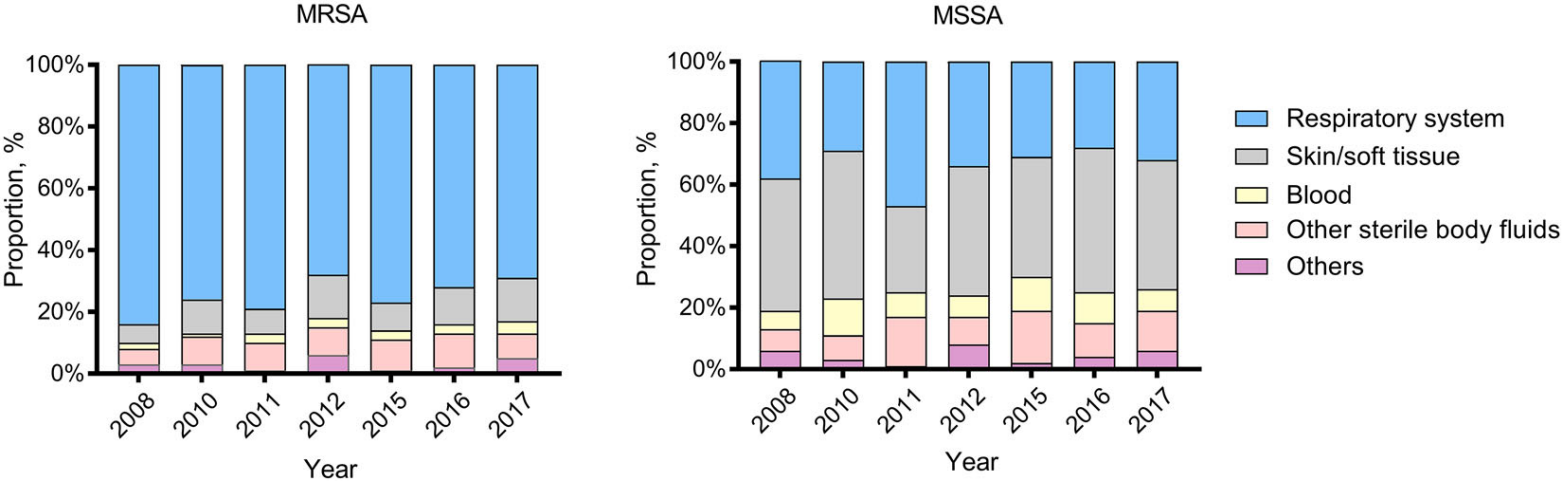
Dynamic changes in the annual proportion of different clinical specimens from which MRSA or MSSA isolates were recovered, 2008–2017.

Despite the gradual decline in *S. aureus* respiratory infection cases, respiratory specimens still constituted the overwhelming majority of specimen sources (76% in 2008 and 52% in 2017, respectively) (Table 1). Bacteraemia occurred with an average frequency of 5% in our study. Of note was the significantly increasing trend in the proportion of SSTI since 2008 (from 12% to 27%, *p* < .01), among which MSSA rates were more prominent relative to other specimen types (Figure 1). However, a striking difference was observed in the prevalence of MRSA among different specimen types. Notably, MRSA rates were highest among isolates from respiratory specimens throughout the past decade (Figure 1).

### Antimicrobial resistance patterns among MRSA and MSSA, and changing resistance trends among overall *S. aureus* isolates, 2008–2017

**Figure 2.**
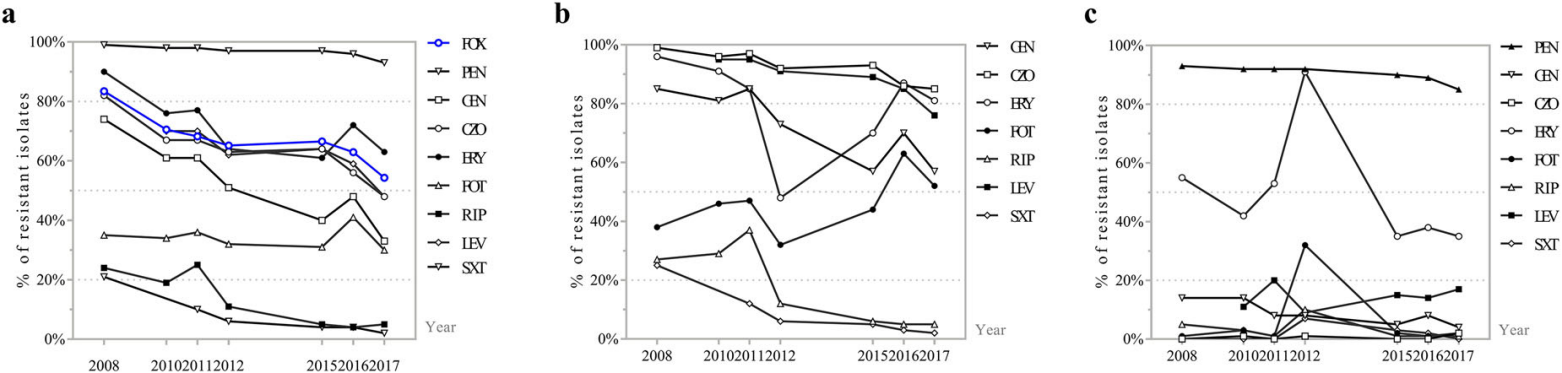
The constantly changing antibiotic resistance profile of (a) Total *S. aureus* isolates, (b) MRSA isolates, (c) MSSA isolates, 2008–2017. FOX: cefoxitin; PEN: penicillin; GEN: gentamicin; CZO: cefazolin; ERY: erythromycin; FOT: fosfomycin; RIF: rifampin; SXT: trimethoprim/sulfamethoxazole; LVX: levofloxacin.

It is notable that a descending trend was observed in the overall MRSA rate, which dropped remarkably from 83.5% (643/770) in 2008 to 54.2% (228/421) in 2017 (*p* < .0001) (Figure 2(a)). The annual resistance rate of *S. aureus* isolates to all antibiotics but fosfomycin decreased significantly (*p* < .05) (Figure 2(a)). Generally, trimethoprim–sulfamethoxazole and rifampin displayed superior antimicrobial activity against both MRSA and MSSA isolates, whereas erythromycin and penicillin exhibited the poorest antimicrobial activity (Table 2).

**Table 2.** Dynamic changes in annual resistance of MRSA and MSSA to several antimicrobial agents from 2008 to 2017 (rate of resistance, %).

Antimicrobial agent	2008	2010	2011	2012	2015	2016	2017	*P*-value^a^								
	MRSA	MSSA	MRSA	MSSA	MRSA	MSSA	MRSA	MSSA	MRSA	MSSA	MRSA	MSSA	MRSA	MSSA	MRSA	MSSA
PEN	100%	93%	100%	92%	100%	92%	100%	92%	100%	90%	100%	89%	100%	85%	/	**0.0398**
GEN	85%	14%	81%	14%	85%	8%	73%	8%	57%	5%	70%	8%	57%	4%	**<0.0001**	**0.0026**
CZO	99%	0%	96%	1%	97%	0%	92%	1%	93%	0%	86%	0%	85%	2%	**<0.0001**	0.3159
ERY	96%	55%	91%	42%	85%	53%	48%	91%	70%	35%	87%	38%	81%	35%	**0.0014**	**0.0012**
FOS	38%	1%	46%	3%	47%	1%	32%	32%	44%	2%	63%	1%	52%	2%	**0.0056**	>0.9999
RIP	27%	5%	29%	3%	37%	1%	12%	10%	6%	1%	5%	1%	5%	1%	**<0.0001**	0.0831
SXT	25%	0%	/	12%	0%	6%	7%	5%	3%	3%	2%	2%	0%	**<0.0001**	0.8644	
LVX	/	95%	11%	95%	20%	91%	9%	89%	15%	85%	14%	76%	17%	**<0.0001**	0.5392	
LZD	0%	0%	0%	0%	0%	0%	0%	0%	0%	0%	0%	0%	0%	0%	/	/
VAN	0%	0%	0%	0%	0%	0%	0%	0%	0%	0%	0%	0%	0%	0%	/	/

Notes: Blank cells indicate not tested. MRSA: methicillin-resistant *S. aureus*; MSSA: methicillin-susceptible *S. aureus*; PEN: penicillin; GEN: gentamicin; CZO: cefazolin; ERY: erythromycin; FOT: fosfomycin; RIF: rifampin; SXT: trimethoprim/sulfamethoxazole; LVX: levofloxacin; LZD: linezolid; VAN: vancomycin.

^a^*P*-value by chi-square test for trend, for detecting whether the increasing or decreasing trend in resistance rates among MRSA or MSSA existed over the past decade. A *p*-value below .05 was considered to be significantly different and is indicated in bold face.

The antimicrobial resistance pattern of MRSA was still obviously different from MSSA, as resistance rates of MRSA to most antimicrobials were commonly higher relative to MSSA (Table 2). A significantly growing trend in the resistance rate of MRSA to fosfomycin was observed, whilst the annual resistance profile of MRSA isolates demonstrated significantly decreasing trends to all other antibiotics tested but penicillin, vancomycin, and linezolid; neither vancomycin- or linezolid-resistant isolates nor penicillin-sensitive MRSA isolates were detected in this study (Table 2 and Figure 2(b)). Unlike the multidrug-resistant MRSA isolates, MSSA isolates maintained a high degree of susceptibility to most antimicrobial agents, and maintained high-level resistance mainly to penicillin and erythromycin (Table 2 and Figure 2(c)).

### Shifts in the molecular epidemiology

**Figure 3.**
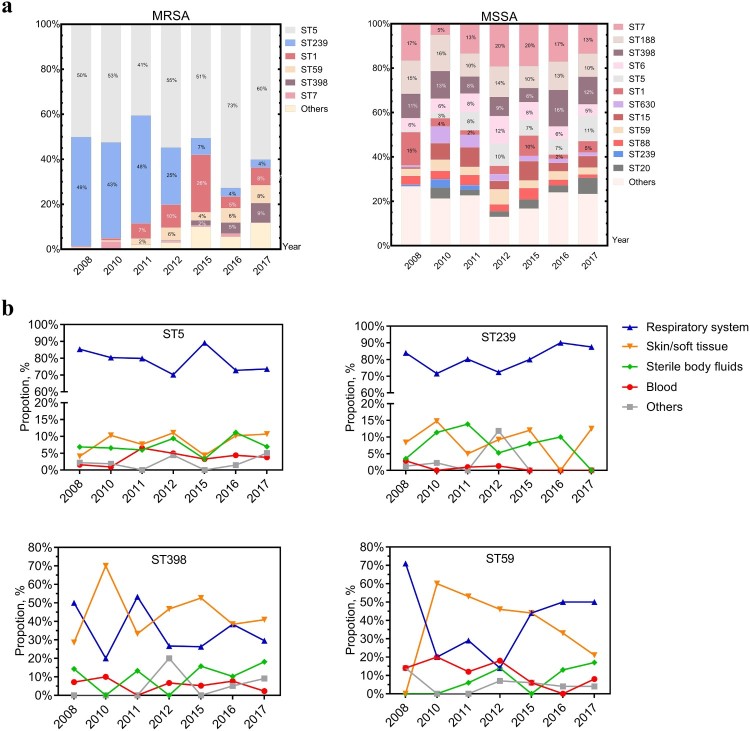
Genotypes and prevalence of certain *S. aureus* clones from different clinical specimen of inpatients, 2008–2017. (a) The dynamic changes of the percentage of MRSA or MSSA clones recovered from the indicated year. Blocks are coloured to reflect the proportion of corresponding sequence type. (b) Prevalence of the epidemic HA-MRSA clones, ST5 and ST239, and the increasingly emerging dominant CA-MRSA clones, ST398 and ST59 among different clinical specimens.

With new sequence types (ST) constantly emerging, a total of 72 ST types was found. MRSA isolates once were dominated by both ST239 and ST5 clones between 2008 and 2011, but exhibited a dramatic decline in ST239 clones from 41% in 2008 to 2% in 2017 (*p* < .0001) (Table 3). ST5, whose proportion didn’t significantly change (from 42% to 38%, 2008–2017) (Table 3), represented the major clone among MRSA isolates (Figure 3(a)). With regard to associated *spa* types, the ST5-t2460 clone that was introduced in 2011 increased significantly to 17.3% in 2017 (*p* < .0001) and superseded the ST5-t002 clone that previously predominated but was significantly (*p* < .0001) reduced from 40% in 2008 to 12.8% in 2017. Interestingly, the ST5-t311 clone had been trending to peak in 2016 but sharply reduced to 2.6% in 2017; a similar pattern was also observed with the ST1-t321 clone (Table 3). A slight but significant increase was also observed among ST398 and ST59 clones (*p *< .0001), two of the most dominant highly virulent CA-MRSA clones (Table 3). MSSA isolates displayed greater genetic diversity than MRSA isolates (Figure 3(a)). The genotypic composition profile remained similar among MSSA isolates over the past decade, generally with only a significant increase in the proportion of ST5-MSSA clones (*p *< .05). Additionally, the isolation rate of both ST239 and ST5 were highest amidst respiratory infection over the decade. ST398 was primarily found in both respiratory infection and SSTI, but the proportion of ST59-caused SSTI showed a trend of gradual decrease (*p *< .0001) (Figure 3(b)).

**Table 3.** Percentage of major clone complex (CC), sequence types (ST) and *spa* types among *S. aureus* isolates, 2008–2017.

CC	ST	*Spa* type	2008	2010	2011	2012	2015	2016	2017
**CC5**	ST5	t002	**40.0%**	**31.6%**	**26.3%**	**23.6%**	**12.3%**	**18.3%**	**12.8%**
		t601	1.2%	0%	0.2%	0%	0%	0.2%	0%
		t548	0.4%	1.1%	0.3%	1.1%	1%	1.4%	1.4%
		t214	0.1%	1.4%	0.5%	0.4%	1%	0.5%	0%
		t2460	**0**	**0**	**1.3%**	**10.4%**	**11.7%**	**11.3%**	**17.3%**
		t311	**0**	**0**	**1.2%**	**2.6%**	**8.4%**	**12.0%**	**2.6%**
		Others	0.3%	3.9%	0.5%	1.1%	1.6%	4.7%	3.6%
		***Subtotal***	***41.9%***	***37.9%***	***30.3%***	***39.2%***	***35.9%***	***48.4%***	***37.8%***
**CC8**	ST239	t030	**20.3%**	**18.8%**	**23.0%**	**10.8%**	**1.9%**	**0.9%**	**1%**
		t037	**18.4%**	**9.9%**	**8.6%**	**5.0%**	**2.5%**	**0.7%**	**0.5%**
		t421	1.4%	1.8%	0.2%	0%	0%	0%	0%
		Others	0.5%	0.7%	1.5%	0.6%	0.4%	0.7%	0.5%
		***Subtotal***	***40.6%***	***31.2%***	***33.2%***	***16.5%***	***4.9%***	***2.3%***	***1.9%***
	ST630	t7291	0%	0	0.8%	0.9%	0.2%	0%	0%
		t4549	0%	0.4%	0.3%	0.6%	2.3%	0.5%	1%
		t2196	0%	0.7%	0%	0%	0%	0.2%	0.2%
CC7	ST7	t091	2.6%	2.5%	2.5%	5.4%	3.3%	5.4%	4.5%
		t796	0%	1.1%	1.0%	1.7%	1.0%	0.5%	0.7%
CC1	ST1	t127	2.5%	1.8%	0.7%	1.7%	1.8%	0.5%	2.6%
		t321	0%	0%	3.9%	6.1%	17.9%	3.3%	3.6%
CC188	ST188	t189	2.5%	3.9%	3.5%	4.5%	3.3%	4.2%	4.3%
CC6	ST6	t701	0.9%	1.1%	2.3%	3.9%	2.7%	1.4%	1.9%
**CC59**	ST59	t437	**0.6%**	**1.1%**	**1.3%**	**4.5%**	**2.7%**	**4%**	**2.9%**
		Others	0.4%	0.7%	1.5%	1.5%	0.8%	1.4%	2.9%
		***Subtotal***	**1.0%**	**1.8%**	**2.8%**	**6.0%**	**3.5%**	**5.4%**	**5.8%**
**CC398**	ST398	t034	**0.5%**	**1.1%**	**0.8%**	**1.3%**	**2.7%**	**5.2%**	**4.8%**
		t571	0.5%	1.8%	0.8%	1.1%	0.2%	1.4%	1.2%
		Others	0.8%	0.7%	0.8%	0.9%	0.8%	2.6%	4.5%
		***Subtotal***	**1.8%**	**3.6%**	**2.4%**	**3.2%**	**3.7%**	**9.2%**	**10.5%**
CC15	ST15	t084	0.1%	2.2%	1.3%	1.3%	1.6%	0%	1.2%
		t085	0%	0%	1.5%	0%	0%	0.2%	0%
Others	8%	16%	18%	12%	21%	28%	36%		

Note: Genotypes with frequency less than 0.5% every year are not listed; significantly increasing or descending trends tested by chi-square test (*p *< .05) are highlighted in bold font.

### The genetic backgrounds associated with the changes in the observed resistance phenotypes

**Figure 4.**
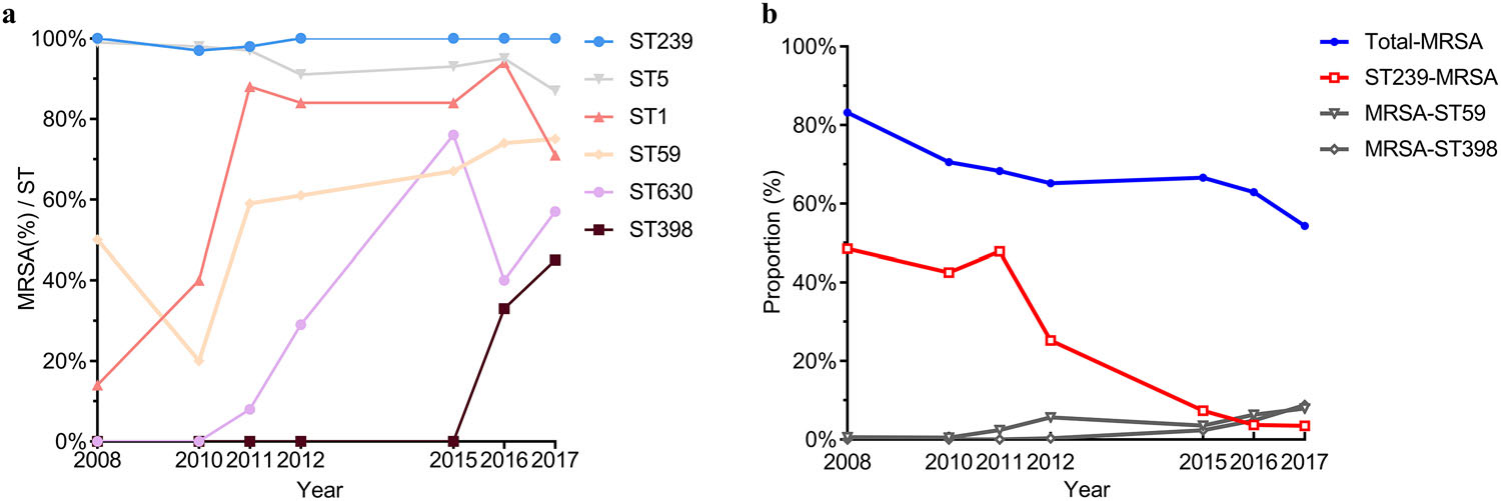
Genotypes and prevalence of certain *S. aureus* clones from different clinical specimen of inpatients, 2008-2017. (a) The dynamic changes of the percentage of MRSA or MSSA clones recovered from the indicated year. Block sizes reflect the proportion of corresponding sequence type. (b) Prevalence of the epidemic HA-MRSA clones, ST5 and ST239, and the increasingly emerging dominant CA-MRSA clones, ST398 and ST59 among different clinical specimens.

The resistance patterns vary substantially between diverse genotypes. MRSA isolates usually resistant to multiple antibiotics showed distinguishable genotypic composition (Figure 3(a)). The vast majority of *S. aureus* ST239 clones over the past decade were MRSA (97–100%) (Figure 4(a)) but a less frequent annual isolate rate of MRSA was observed among certain clones, such as ST188, ST15, and ST6 (5–11%, 0–9%, 0–18%, respectively, data not shown). Additionally, 14% of ST1 clones were found to be MRSA in 2008; this proportion reached a peak of 94% in 2016, demonstrating a drastically increasing trend in ST1-MRSA clones (*p *<* *.0001) (Figure 4(a)). Likewise, the proportion of ST630-MRSA clones significantly mounted from 0% (2008) to 57% (2017) (*p *< .0001) (Figure 4(a)). Notably, the proportion of MRSA among ST398 clones reached to 45% in 2017 from 0% in 2015; an increasing proportion of MRSA among ST59 clones was also observed (from 50% to 75%, 2008–2017, respectively, *p *<* *.0001) (Figure 4(a)).

However, the observed significantly descending trend in MRSA prevalence during the study period of 2008 through 2017 was positively correlated with the marked decline of ST239-MRSA (*p* < .05) (Figure 4(b)). In other words, the decreased prevalence of MRSA was, to some extent, attributed to the disappearance of ST239-MRSA clones (from 41% in 2008 to 2% in 2017, *p *<* *.001).

## Discussion

With the emergence of MRSA worldwide, effective interventions have played a vital role in preventing transmission and development of resistance [13,14]. Although a marked decrease in the prevalence of MRSA among Chinese hospitals within recent years has been already reported by the China Antimicrobial Surveillance Network (CHINET) [15], and evolutionary changes of MRSA have been documented in China [2], there have been few reports, however, associating the decrease in prevalence of MRSA with clonal shifts. In the present retrospective study, *S. aureus* isolates recovered from 2008 to 2017 in a comprehensive teaching hospital in Shanghai were characterized and subjected to genetic multi-locus sequence typing and *spa* typing. Taking into consideration the decreasing prevalence of MRSA together with the apparent shifts in clonal composition among MRSA isolates, we attributed the decline in the prevalence of MRSA to the significant decrease of the previously dominant ST239-MRSA clones.

The Asian-dominant HA-MRSA ST239 clones have persisted and adaptively evolved in hospital environments for decades [16,17], and have accounted for the overwhelming majority of HA-MRSA across not only China but other Asian regions as well [18, 19]. However, the molecular characterization in the present study disclosed that three previously predominant clones across China were on the wane, as we noticed the remarkable decline in both ST239-t030 and ST239-t037 clones, both of which accounted for the large proportion of all isolates across China [9, 20]. Although the proportion of another previously predominant HA-MRSA ST5 clones has not changed significantly in general, the concomitant decrease in the numbers of the ST5-t002 clone was detected. It is noteworthy that ST5-t2460, which had more than doubled since it was introduced in 2010 and rapidly increased in prevalence since then, was progressively replacing the previously dominant clones of ST239-t030 and ST239-t037, and accounted for 31% of all MRSA isolates in 2017. Little information is available about the average percentage across China and the evolutionary process of the ST5-t2460 clone; of particular interest was the aborted surge in the ST5-t311 clone found in the present study, but the successful dominance of the ST5-t311 clone has been documented in other Chinese hospitals [21]. Therefore, whether the ST5-t2460 clone has been conferred more competitive advantages in the hospital setting remains bewildering. Even though the prevalence of ST5-t2460 is currently not too high, the tendency of this multidrug-resistant clone to supersede other clones warrants its continuous monitoring in the coming years.

A statistically significant decrease in the annual MRSA isolation rate, as a proportion of the total *S. aureus*, was noted. Since the proportion of ST239-MRSA clones was reduced dramatically, a positive correlation between the substantial reduction in ST239-MRSA clones and the decreasing prevalence of MRSA was observed. However, previous studies have demonstrated that the frequent clonal replacement of MRSA, ST239-MRSA-SCC*mec*III has been replaced by both ST5-MRSA-SCC*mec*II and ST228-MRSA-SCC*mec*I in Hungary [22]. In view of the fact that the dominance of limited numbers of pandemic MRSA clones was attributable to their unique abilities to cope with the contemporary clinical environment [23], preventative strategies and antibiotic usage played important role in the changing molecular epidemiology by providing more intense selective pressure to drive competition between clones, thus ST239 clones might be less adaptive and competitive than a growing number of newly emerging clones when confronted a highly selective pressure in health-care settings, probably leading to the marginalization of ST239 clones. HA-MRSA infections are transmitted mainly through hand contact [24] but the hand hygiene programme launched by the hospital of the present study, either by hand washing or hand disinfection prevention, remained the same throughout the study period. The primary therapy for MRSA infection has been consistently according to the IDSA guidelines that recommend vancomycin as the primary option for treatment of MRSA infections [25], while other newly approved anti-MRSA agents including linezolid, daptomycin and tigecycline have established significant roles as first-line agents in selective patients as well during the last decade [26]. Though antibiotic resistance is vital for pathogens survival in hospitals, no evidence of vancomycin MIC creep phenomenon was found by a systematic review and meta analysis included 29234 *S. aureus* isolates reported in the 55 studies [27]. It’s notable that linezolid was approved for clinical use by China Food and Drug Administration (CFDA) after 2007 as an alternative to treat infections unresponsive to vancomycin, in addition, linezolid has been regarded as superior to vancomycin in both cure rates and eradication rates of MRSA infections [28]. But whether the exposure to linezolid rendered ST239 clones less competitive remains unclear and the mechanisms underlying the failure of ST239 clones within the health-care environment deserve further research.

Community-acquired *S. aureus* ST398 clones have rapidly emerged in recent years [29, 30] though the prevalence of ST398-MRSA in the present study appeared to be low but was increasing. It has been previously revealed that multiple acquisitions of staphylococcal cassette chromosome *mec* (SCC*mec*) by MSSA precursors frequently occurred in ST398-MRSA [31], and more importantly, CA-MRSA ST398 were able to evolve from methicillin-sensitive ST398 without constant genetic alteration in virulence [32]. Coinciding with the increase in ST398 was the increasing emergence of another epidemic lineage of CA-MRSA clones, ST59, which accounted for up to two-thirds of CA-MRSA isolates between 2006 and 2008 [33]. The frequent appearance of CA-MRSA clones entering health-care settings in recent years has been observed in the present study, as reported elsewhere [34]. Acquired mobile genetic elements (MGEs) and several genes mutations that confer vancomycin non-susceptibility have been identified in sequenced ST59 genome [35, 36], suggesting ST59 underwent multiple evolutionary events essential for better adaptability within the hospital. Data from this epidemiological study were not enough to provide relevant information on the reduced survival advantages or dissemination of ST239 clones, thus further study is under way to ascertain evolutionary changes that drove those remarkable clonal shifts, including MGEs (prophages, transposons and plasmids, etc.). Nonetheless, WGS based comparative genomics is not convincing enough to ascertain wherefores that contributed to the disappearance of ST239 and encroachment of ST59 and ST398 due to their inherently disparate genome. Additionally, virulence gene content differed a lot between CA- and HA-MRSA, CA-MRSA clones commonly have high expression of core genome-encoded toxins (e.g. α-toxin, PSM), investigating differentiation in transcriptional profile, alongside phenotypic measurements, is indispensable for giving insights into evolutionary dynamics therefore.

MSSA isolates were still composed of more genotypes than MRSA isolates, suggesting that the MSSA population was more heterogeneous relative to the epidemic MRSA clones and probably had a differential genetic background.

In addition, the changing resistance profile of the MRSA isolates in the present study indicated that the traditional multidrug-resistant MRSA clones became less frequent with the emergence and spread of MRSA clones with susceptibility to gentamicin and other antibiotics. However, strict antimicrobial-use guidelines are still required owing to the multifactorial relationship between antibiotics use and resistance evolution [37].

Regardless of the markedly decreasing prevalence of MRSA in our study, the proportion of MRSA still reached nearly half of all *S. aureus* isolates, below that of many hospitals in China [15]. In light of these facts, the prevention and control of the spread of MRSA remain a crucial and challenging task. The multidrug-resistant ST5-t2460, likely a candidate for the leading clone in the near future, together with the increasing isolation rates of MRSA among ST1 and ST630 clones, were of great concern. Further investigations are still of great importance for monitoring the changing epidemiological trends.
